# Perceptions of chief clinical information officers on the state of electronic health records systems interoperability in NHS England: a qualitative interview study

**DOI:** 10.1186/s12911-023-02255-8

**Published:** 2023-08-12

**Authors:** Edmond Li, Olivia Lounsbury, Jonathan Clarke, Hutan Ashrafian, Ara Darzi, Ana Luisa Neves

**Affiliations:** 1 Institute of Global Health Innovation, National Institute for Health and Care Research (NIHR) Imperial Patient Safety Translational Research Centre, Imperial College London, London, UK; 2 Johns Hopkins Children’s Center, Baltimore, MD USA; 3 Centre for Mathematics of Precision Healthcare, Department of Mathematics, Imperial College London, London, UK; 4 Department of Primary Care and Public Health, Imperial College London, London, UK; 5 Department of Community Medicine, Health Information and Decision, Center for Health Technology and Services Research, University of Porto, Porto, Portugal

**Keywords:** Electronic health records, Interoperability, Patient safety, Health IT, Health policy, Qualitative research, Semi-structured interviews

## Abstract

**Background:**

In the era of electronic health records (EHR), the ability to share clinical data is a key facilitator of healthcare delivery. Since the introduction of EHRs, this aspect has been extensively studied from the perspective of healthcare providers. Less often explored are the day-to-day challenges surrounding the procurement, deployment, maintenance, and use of interoperable EHR systems, from the perspective of healthcare administrators, such as chief clinical information officers (CCIOs).

**Objective:**

Our study aims to capture the perceptions of CCIOs on the current state of EHR interoperability in the NHS, its impact on patient safety, the perceived facilitators and barriers to improving EHR interoperability, and what the future of EHR development in the NHS may entail.

**Methods:**

Semi-structured interviews were conducted between November 2020 – October 2021. Convenience sampling was employed to recruit NHS England CCIOs. Interviews were digitally recorded and transcribed verbatim. A thematic analysis was performed by two independent researchers to identify emerging themes.

**Results:**

Fifteen CCIOs participated in the study. Participants reported that limited EHR interoperability contributed to the inability to easily access and transfer data into a unified source, thus resulting in data fragmentation. The resulting lack of clarity on patients' health status negatively impacts patient safety through suboptimal care coordination, duplication of efforts, and more defensive practice. Facilitators to improving interoperability included the recognition of the need by clinicians, patient expectations, and the inherent centralised nature of the NHS. Barriers included systems usability difficulties, and institutional, data management, and financial-related challenges. Looking ahead, participants acknowledged that realising that vision across the NHS would require a renewed focus on mandating data standards, user-centred design, greater patient involvement, and encouraging inter-organisational collaboration.

**Conclusion:**

Tackling poor interoperability will require solutions both at the technical level and in the wider policy context. This will involve demanding interoperability functionalities from the outset in procurement contracts, fostering greater inter-organisation cooperation on implementation strategies, and encouraging systems vendors to prioritise interoperability in their products. Only by comprehensively addressing these challenges would the full potential promised by the use of fully interoperable EHRs be realised.

**Supplementary Information:**

The online version contains supplementary material available at 10.1186/s12911-023-02255-8.

## What is already known on this topic

Although Electronic Health Record (EHR) deployment in the National Health Service (NHS) has steadily increased, interoperability between healthcare facilities has remained problematic. Despite numerous policy initiatives aimed at tackling this issue, little success has been found in rectifying this issue to improve effective data sharing.

## What this study adds

Our study captured the current state of EHR interoperability and its perceived facilitators and barriers from a unique and often underrepresented perspective: Chief Clinical Informatics Officers (CCIO). CCIOs have noted several facilitators toward interoperability, including the recognition of need from clinicians, expectations from patients, and the inherently centralised arrangement of the NHS itself. CCIOs have also highlighted a range of issues precluding the NHS from achieving greater EHR interoperability, from local issues including poor systems usability and data management, to more systemic ones such as institutional barriers, and financial obstacles. Accumulating EHR experience, coupled with the COVID-19 pandemic, has accelerated a renewed sense of urgency towards prioritising interoperability. Future efforts to improve EHR interoperability should anticipate emerging themes such as patients’ role in their EHR data and facilitating more effective inter-organisational collaboration across the NHS.

## How this study might affect research, practice, or policy

Our study aims to inform on the establishment of more relevant, sustainable and cost-effective approaches to implementing and utilising interoperable EHR systems in the NHS. Targeted health technology policies addressing the identified limitations will help both make existing systems safer and streamline care delivery. Accomplishing this will allow such systems to evolve and better meet the changing healthcare needs confronting the NHS in the coming years.

## Introduction

### Background

Since the early 2000s, electronic health records (EHR) have played an increasingly integral role in the clinical environments of most high-income countries [1–4]. Many of the purported benefits of EHR (*i.e.,* more effective care coordination, communication between providers, lower healthcare costs, improving patient safety), rely upon the effective exchange of information between various systems [5–10].

Interoperability, defined as *‘the ability for clinical data to be shared seamlessly between differing EHR systems without loss of context and for the data to be usable in a coordinated manner to facilitate patient care’*, has continued to be a growing challenge [7, 11, 12]. Due to the inherent technical complexity and nature of the clinical information involved, interoperability between existing systems varies greatly in both availability and sophistication [13, 14].

The Healthcare Information and Management Systems Society (HIMSS) categorises interoperability into three tiers: (1) foundational interoperability, (2) structural interoperability, and (3) semantic interoperability [12, 15–17]. Foundational interoperability describes *‘the inter-connectivity requirements needed for one system or application to securely communicate data to and receive data from another’* [12, 15]. Structural interoperability refers to information exchange between health information technology (HIT) systems at the data field level, made possible using common data formats, syntax, and organisational standards [12, 17]. Semantic interoperability, the most complex of the three levels, describes the use of open standards (*e.g.*, Fast Healthcare Interoperability Resources (FHIR) or Health Level Seven (HL7)) for codifying data elements (*i.e.*, content, terminology, and security to enable a shared understanding of clinical data) [7, 12, 15]. It should be noted, however, other definitions which stratify levels of interoperability in greater detail (such as technical, syntactic, pragmatic, dynamic, conceptual, structural, functional, and semantic interoperability), have also been proposed in recent years [18].

There are various approaches globally to improve interoperability, with varying levels of success. In the United States, the 2009 Health Information Technology for Economic and Clinical Health (HITECH) Act and accompanying Meaningful Use programme aimed to incentivise EHR integration and the establishment of more robust health information exchanges [5, 19–21]. In the subsequent years, for-profit healthcare delivery systems (*i.e.,* Kaiser Permanente), successfully introduced integrated HIT across hospitals, community-based clinics, pharmacies, and laboratories [22–24]. Similar efforts were undertaken in Canada, Demark, and Saudi Arabia [4, 24, 25].

In the United Kingdom, the 2002 National Programme for IT (NPfIT) attempted to introduce a centralised EHR system with integrated electronic patient records, appointment scheduling, patient referrals, and prescription renewal systems by 2010 [2, 10, 26–28], but the initiative was eventually cancelled in 2011, due to financial and implementation issues [10, 24, 27, 28]. The UK continued funding similar efforts aimed at establishing integrated EHRs [29, 30] but, to date, these efforts resulted only in the establishment of modest regional networks with limited interoperability [24, 27, 28, 31].

Research on EHR interoperability has traditionally been US-centric and focused on provider perspectives [29, 32–35]. Potential benefits identified included greater data accuracy and easier access to information, and improved efficiency and timeliness of care [19, 35, 36]. Potential barriers have also been reported, including high costs, organisational barriers to change, mixed provider satisfaction, technological hurdles, and data overload for users [36–38]. Less attention has been paid to investigating the pragmatic challenges to interoperability from the perspective of other stakeholders in the health system (*i.e.,* healthcare administrators), as well as their consequences on patient safety. In the UK, Chief Clinical Informatics Officer (CCIO) is a senior role which *‘provides leadership and management of ICT (information and communications technologies) and information development activity to support the safe and efficient design, implementation and use of informatics solutions to deliver improvements in the quality and outcomes of care’* [39–42]. CCIOs usually are clinicians (*i.e.,* doctors, nurses, pharmacists) who also possess clinical informatics expertise, training, and experience and are involved in introducing, using, and maintaining HIT systems. As such, ascertaining their unique perspective regarding the present state of EHR interoperability in the NHS would be beneficial to highlight administrative and grass-roots level interoperability challenges and describe how they impact patient safety in NHS settings.

### Aim and objectives

This study aims to gain a better understanding of EHR interoperability and its impact on patient safety from the perspective of CCIOs. Specific objectives include:

1. Capture the perceptions of CCIOs regarding the current state of EHR interoperability.
2. Assess its perceived effect on patient safety.
3. Investigate facilitators and barriers to achieving interoperability.
4. Explore perceptions on how the evolution of EHR interoperability would improve patient safety in the coming decade.

## Methods

Semi-structured, in-depth, 1:1 online interviews were utilised due to its ability to explore participants’ thoughts, feelings and beliefs about a given topic, delving into their personal experiences [43]. Interviews were conducted between November 2020 – October 2021 using a standardised topic guide (Supplement 1) and lasted 45–60 min. The interviews were digitally recorded and transcribed verbatim. No repeat interviews were conducted.

### Study population

The study population of interest were CCIOs based in NHS England primary and secondary healthcare facilities, from various clinical backgrounds (i.e., doctors, nurses, pharmacists). Participants must have had at least one year of experience working with EHR systems in NHS settings in England. Only English speakers were included.

### Participant recruitment

Participants were identified using the NHS Digital Academia Alumni network, and snowball sampling was subsequently used to increase the sample. CCIOs who met the inclusion criteria were invited to participate via email.

### Data analysis

Audio recordings were transcribed and thematically analysed by two researchers independently (EL, OL). The analysis was both deductive and inductive in its approach. Regular meetings between the lead researcher and other members of the research group took place to assess data saturation, ensure coding quality, and refine the codes and subthemes. Microsoft Excel and Miro were used to organise codes and resultant themes.

## Results

**Fig. 1:**
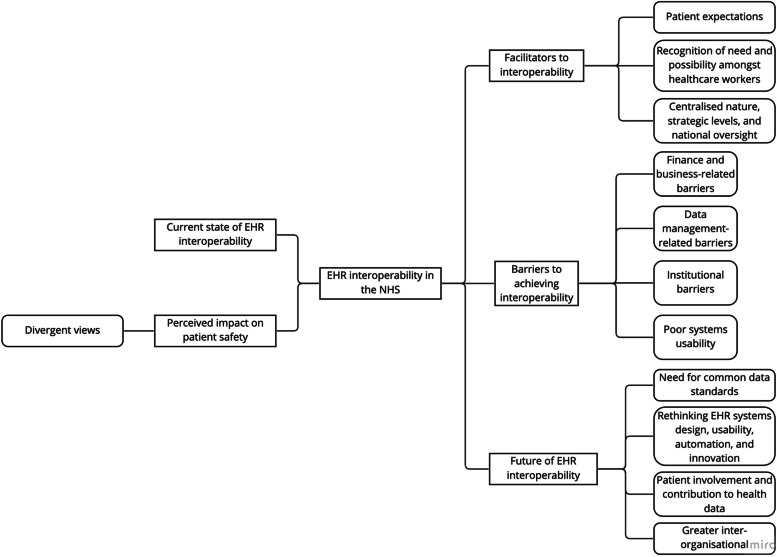
Mapping of prominent themes identified

A total of 15 NHS England CCIOs were interviewed. For characteristics of the study participants, please see (Table 1). Five main emergent themes and their respective subthemes were mapped (Fig. 1).

**Table 1:** Study participant characteristics

Characteristics	*n* (%)
**Role**	
Physicians	11 (73.3%)
GP	3 (20%)
Specialist	8 (53.3%)
Other healthcare professionals	4 (26.6%)
Medical microbiologist	1 (6.7%)
Physiotherapist	1 (6.7%)
Emergency medical technician (EMT)	1 (6.7%)
Unknown	1 (6.7%)
**Years of CCIO experience**	
1–2	8 (53.3%)
3–4	3 (20%)
5 +	4 (26.6%)
**NHS England regions**	
North East and Yorkshire	1 (6.7%)
North West	5 (33%)
East of England	1 (6.7%)
London	3 (20%)
Midlands	1 (6.7%)
South East	2 (13.3%)
South West	1 (6.7%)
Unknown	1 (6.7%)
**Healthcare facility type**	
Hospital-based	13 (86.6%)
Community-based	1 (6.7%)
Mixed	1 (6.7%)

### Perceptions on the current state of EHR interoperability in the NHS

Amongst participants, there is a lack of consensus regarding what interoperability entails (Table 2). For some, interoperability meant simply being able to access a recent GP letter scanned in PDF form; for others, it meant being able to trace a patient’s trajectory of care through their last hospital stay, including clinical notes and laboratory results.

**Table 2:** Current state of EHR interoperability

“You can't rely on it having a complete set of data, so you still need to talk through with the patient, but it does alert you to things which are there. There are still risks. You still need to check that medications are correct because there's holes in the medication record there, because that's just the GP record, but it doesn't tell you anything about someone who might be under the drug and alcohol service for example, because that's a different service and doesn't feed into our record. It doesn't tell you if a secondary care physician, even in the same hospital, has given them medication on an outpatient discharge or anything like that, so the risks are more about the data that isn't there, and not being aware that that data isn't there.” – Participant 10
“Yes, so we have lots of sticking plaster interoperability. […] I can view some of your healthcare information online. I may see some of your primary care information, I may see that you've had an episode of care at another site. I wouldn't be able to see the details of it. So, we're creating ways to view information, but it's not interoperable. We're not sharing the information. That's pretty much the state of play. It's very limited data transfer.” – Participant 12
“We are in a position of the absence of information is not instructive. It didn't mean you didn't have your blood pressure checked, it just means that the GP might not be sharing that yet, or something like that. […] I don't know what I don't know. There is no meaningful information sharing between secondary care sites.” – Participant 12
“If we had a fully national interoperable approach to data, then I would be able to change the way I work, because I would be able to look at your record, in confidence, and know that is a single version of the truth. I therefore do not need to do all these things. At the moment, because I do not have that trust, and no one has that trust, we look at it, but we still do the full A to Z assessment.” – Participant 12
“I think if you were to say the word 'interoperability' to most clinicians, they would have no idea what you meant. Certainly, the interoperability of data, if you took it away, they would certainly understand what you meant by it because they would suddenly find that the electronic health record, without that interoperability—the order communications and results section of our EHR would disappear.” – Participant 13

Information sharing between primary and secondary care, and between different clinical specialties remains especially problematic, particularly concerning sensitive information (*e.g.*, mental health conditions). The level of data transferrable or accessible also varies greatly between medical staff and allied health teams. Limited data exchange abilities result in substantial patient data fragmentation, data quality issues, and suboptimal clinical workflows.

CCIOs reported that when procuring EHR systems, the importance of interoperability with other healthcare facilities was often only recognised after implementation.

### Perceived impact on patient safety

Many participants noted that limited EHR interoperability often contributed to longstanding issues concerning EHR data quality which negatively impacted patient safety (Table 3).

**Table 3:** Perceived impact on patient safety

“There are probably three or four hospitals that patients might go to that I won't be able to see anything. How it impacts, some of it is small things like having to repeat blood tests to make sure that somebody's safe. Sometimes it's […] repeating scans that you otherwise wouldn't have done if you'd have known that result… […]” – Participant 1
“So I think it's [poor interoperability] a major problem actually. So almost any sphere where you need patient information, there is a safety element to that and anywhere where that information is getting summarised, or handed over, or transcribed, or recoded, there's an element of error in all of that, and an element of loss in all of that. So almost any safety element is magnified by that lack of interoperability. The classic and most obvious one is around medication. So quite often when you get that transfer, you get a list of current medication. So for us quite often we know what medication somebody had before they went into hospital and what they came out on, but what you don't get so much of is why those changes were made. What was stopped, why it was stopped, what was restarted, why was it restarted, why the changes were made. So you don't know if they were to do with a patient factor or a system factor. So automatic switches for cost reasons. You don't know if the change was for a cost reason or because of a patient factor such as side-effects. Anything where you've got to hand over of a task as well. So it relies on someone reading a letter, so there's a manual process in reading it, in picking out the task, and ensuring the task will happen. Whereas if you're using the same system, actually the task comes and actually sits in an inbox for you and it doesn't go away until you action it. So again, it's quite easy to miss actions passing from one organisation to another or follow up as a result of those actions.” – Participant 5
“But now we've got the GP shared record, I can get an updated list of their previous attendance, recent attendances, medications, allergies, all from just sharing that record. So that's got to be beneficial for patient safety because if they're presented unconscious, but I know who they are, what their NHS identifiable is, who their record is. I can look in their GP record and look for allergies, what medications have been on recently, which may have caused them to deteriorate and also about end-of-life care wishes. […] Because we can share information about end of life care then I can maybe provide more appropriate care for the individual at the end of life, whereas before, if they've been very sick, we may not know what their end of life wishes are, but because we're sharing that information now, then we can provide more appropriate care targeted.” – Participant 7
“We have so many care systems which are now, rely on having information from the shared care record and they can make better decisions if they can see what's in the shared care record, so if it's not there then it's more risky. […] You can't rely on it having a complete set of data, so you still need to talk through with the patient, but it does alert you to things which are there. There are still risks. You still need to check that medications are correct because there's holes in the medication record there, because that's just the GP record, but it doesn't tell you anything about someone who might be under the drug and alcohol service for example, because that's a different service and doesn't feed into our record. It doesn't tell you if a secondary care physician, even in the same hospital, has given them medication on an outpatient discharge or anything like that, so the risks are more about the data that isn't there, and not being aware that that data isn't there.” – Participant 10
“We have had zero interoperability and now we have some. So I think my workflow, and all of my colleagues', accounts for that. Which is when I see you, whatever information I have, I will review with the patient, and I will fill in the blanks. So, I do not think the lack of interoperability and information impacts safety directly, for our service. I think it causes an efficiency problem. […] I think in terms of chronic disease management, I do not see huge safety risks. […] I struggle to think of direct harms that occur due to a lack of interoperability. I can see delays in ongoing care optimisation. I can see efficiency losses and I can see degradation to the staff and the patient experience.” – Participant 12
“How else, in terms of safety? I think the other thing is not having clarity on the management plan—so delays in information which comes through from hospital, from secondary care. […] We typically get patients coming back from a hospital appointment going, 'A doctor's given me a new yellow pill: can you prescribe it?' 'What is it?' We don't know. There's quite a number of issues in terms of medication errors.” – Participant 14

One shortcoming resulting from the lack of interoperability is the inability to easily access and transfer data into a unified source; participants perceived this to be detrimental to patient safety as they do not have clarity regarding the patient’s overall clinical status with which to base their clinical decision-making on. Participants also noted that lack of interoperability results in data fragmentation in several sources, and information being presented in a format that is not easily accessible, and in non-standardised formats.

Together, these aspects culminate suboptimal care planning and coordination (*e.g.,* end-of-life care, do-not-resuscitate orders), and increased administrative workload. A frequently cited example is the difficulty of determining an accurate list of the patient’s most current medications or life-threatening allergies, particularly during transitions of care despite knowing the information is likely available elsewhere or with another provider along the patient’s care pathway.

To mitigate these patient safety issues resultant at least in part due to poor interoperability, clinicians often had to duplicate their efforts (*i.e.,* corroborate information with patients, over-reliance on patient recollection, triangulation of information from different sources). Participants also reported that clinicians often acted more defensively (*e.g.*, ordering duplicate diagnostic investigations) in fear of missing pertinent information and potentially leading to patient harm, for which they could be liable.

### Divergent views

One participant noted that while the lack of EHR interoperability did result in him needing to search for missing information, he saw this as merely an inconvenience, but not a patient safety concern overall. However, the participant also acknowledges that this view likely differs depending on the provider’s training/specialty, with specialists (*e.g.,* A&E, medicine subspecialities) more inclined to repeat investigations on admission due to their accessibility in the hospital, compared to GPs in community clinics. Other participants questioned how useful EHR interoperability may be in practice when they often still prefer to refer to their own notes as a ‘trusted source’.

### Facilitators of EHR interoperability

Study participants highlighted several facilitators to achieving greater EHR interoperability, which can be broadly organised into three subthemes (Table 4): (1) recognition of need and possibility amongst healthcare workers (2) expectation from patients, and (3) centralised nature, strategic levers, and national oversight.

**Table 4:** Facilitators of EHR interoperability

**Subtheme 1: Recognition of need and possibility amongst healthcare workers**
*“When you roll out an electronic health record, they don't actually really particularly think about interoperability with, say, a GP system. I think it's only as you progress through the use of health records that you begin to say, 'Well, actually, that will be really useful for me to have.”* – Participant 4
*“As staff have moved and worked around different areas and they've seen the gradual adoption of electronic patient records, the logical question which everybody asks now is, well why can't I? Why can't I see that? Why can't I get that information? So the clinical narrative is changing. The clinical expectation is changing. I think that will become a more powerful lever than it has been in the past. If there is no clinical drive for something, from your users, it is very easy to discount it as a need.”* – Participant 12
*“We created the population analytics platform quite quickly to support COVID-related population health. That was achieved. That's something that we've been talking about for years and then it actually happened in the space of about two months.”* – Participant 13
*“Finally, because the market has been very immature in the past, clinicians have focused on the localised benefits, rather than the systematic benefits, because that is the world that we live in. If everyone else is on paper, right, what can I do with this here for me now. I think as staff—from trainees to senior roles—as staff have moved and worked around different areas and they've seen the gradual adoption of electronic patient records, the logical question which everybody asks now is, well why can't I? Why can't I see that? Why can't I get that information? So the clinical narrative is changing. The clinical expectation is changing. I think that will become a more powerful lever than it has been in the past. If there is no clinical drive for something, from your users, it is very easy to discount it as a need.”* – Participant 12
**Subtheme 2: Expectations from patients**
*“Patients just think their information is all linked up and it's all there or we can access it, and we can access the GP record, and we'll write to the GP and things will be updated. They make these assumptions. If only they knew the truth sometimes!”* – Participant 8
*“From the patient's point of view as well, a lot more power is going to go to the patient's side. They're going to expect doctors to know about them. They expect them to know. We have a system, quite a few hospitals use it, where it's a bit like TripAdvisor really for doctors where patients can write an online review anonymously about a doctor they've seen, or a hospital they've been to or a clinic they've been to.”* – Participant 6
*“We typically get patients coming back from a hospital appointment going, 'A doctor's given me a new yellow pill: can you prescribe it?' 'What is it?'”*—Participant 14
**Subtheme 3: Centralised nature, strategic levers, and national oversight**
*“I think probably UK has got the best opportunity, or the right levers in place, to produce a good go at interoperability because of the National Health Service. I don't believe any other service is as national as the National Health Service. I think insurance, you know, different models of care, delivery of care, and funding of care does potentially cause bigger challenges of commercial interests where the NHS should, if used properly, should be able to avoid a lot of those. I don't think that we should be looking elsewhere for solutions.”* – Participant 2
*“Given that the NHS is pretty self-contained, I would say, even if it has to have foreign vendors for its software, I think it would make more sense to me for it to be mandated that it has to conform to some standards that are set here rather than global standards. I don't think you're going to get global standards in EHRs any time soon.”*—Participant 6
*“Now I am definitely more optimistic because we've got really good national drivers… like healthcare record exemplar programmes. We're seeing in ICS (integrated care systems) objectives it's part of strategies I think at a local level and [at an organizational level]. Interoperability is on our roadmap. We are committed to making this happen for our clinical workforce and the people that use our services. There's a greater movement and dialogue around this, whether it's on national forums and conferences.”* – Participant 11
*“I think that actually workflows across different Trusts are very similar. Whether they're exactly the same or not, they're very similar, and you could have everybody work in the same way.”* – Participant 1

#### Recognition of need and possibility amongst healthcare workers

Participants noted that COVID-19 accelerated the deployment of digital solutions (*e.g.,* non-paper-based tools), exposing some of the tangible benefits of interoperability and raising awareness on the topic. In the past, frontline workers prioritised EHR functions that would influence their own workflows without acknowledging their impact on larger interoperability initiatives. Working across various organisations has highlighted the need to think holistically about interoperability and prompted workers to reflect on its state at their own organisations.

#### Expectation from patients

As a result of poor EHR interoperability, healthcare providers are often less familiar with their patient’s information than what patients typically expected. This required providers to rely on patient-provided information and thus potentially negatively impact patient satisfaction and trust. Participants acknowledged that unmet patient expectations are strong motivators for efforts to improve interoperability.

#### Centralised nature, strategic levers, and national oversight

The UK was perceived to be ideally positioned to be a global leader in healthcare interoperability because of its centralised organisational structure and single organisational identity as the NHS. As workflows are relatively similar between organisations, there is increased potential for standardisation of data handling processes in pursuit of wider interoperability. Participants noted that interoperability is increasingly prioritised for discussion in multiple forums.

### Barriers to achieving EHR interoperability

Barriers identified can be organised into four subthemes (Table 5): (1) systems usability, (2) institutional, (3) data-related, and (4) vendor/finance-related barriers.

**Table 5:** Barriers to achieving EHR interoperability

**Subtheme 1: Poor EHR systems usability**
*“So I think it's worth calling out that most systems were never designed with interoperability in mind, so when you retrofit interoperability inevitably there are going to be some major challenges.” –* Participant 2
*“Most people either don't know to look in there for information, can't be bothered because it's an extra system on which to log into’ so that's fairly moderately penetrated I would say probably.”* – Participant 2
*“We have single sign on in our practice and in the hospital, since they're in their own record for the Trust, they just click on a button and it brings, it automatically signs them into the shared care record, so that makes it easy. The login process is easy, it's not a problem. Hinders are generally when you can't log on, or you have to have a separate password to log on somewhere else.”* – Participant 10
*“The immediate one for us is to get social care on, because that's the one bit we really don't have, but I think they're on board. Mental health is a different issue, and I don't quite know why we can't get them engaged. They seem to be much more worried about sharing their data.”* – Participant 1
*“For us, it's finding a balance between structuring data and making it easy to enter. There's a balance between having structured data that people are prepared to reuse, because they trust, and then finally, theirs is ultimately a system challenge. We've got lots of stuff that's been coded in different ways and undertaking the exercise to harmonise that to one version of the truth, without a lot of data loss or re-work, I think is a challenge.”* – Participant 12
**Subtheme 2: Institutional barriers**
*“It's more a verbal networking, but there doesn't seem to be a way of viewing what projects are ongoing or what stage they're at, who's involved with them, who to connect to, to talk about them. Or if you see a certain issue, or if you do this, then you're taking this out, and did you realise our workflow was dependent on that? So that kind of transparency I don't think is there.”* – Participant 10
*“I think that it's a big issue. One of your barriers which you probably want to talk about is communication between different organisations. […] The other problem is I think’ you don't always know what other organisations are already doing. The projects aren't being, aren't transparent enough, an’ you can't see what the user requests are for each project, what's their expectation of how it should work, and there's probably a significant overlap which most, a lot of organisations could, are just duplicating work.”* – Participant 10
*“The local example, [trust name], massive hospital, they're spending £100 million, over ten years, on [EHR system name]. The solution to interoperability, moving forwards for us, is well you guys could buy it. So they've decided that that's the course of action. The Greater Manchester may be different because Greater Manchester is a highly politicised environment, ever since devolution, it's caused lots of interesting politics. I think people say well look, we are doing this and if you want to play, you play our way. So there's a bit of a power struggle vibe.”* – Participant 12
*“I think firstly, the business case process doesn't support or reward it. So for me saying, I will spend extra time or money, or what have you, to ensure that I can share my records with another hospital, no. My finance director and my board, they might understand why I'd want to do it, but they will not understand why we should pay for it. […] So I'm going to deploy an EPR and I want to make sure the guys at [name] can see everything that the’ want. That's going to cost me. That's going to benefit staff at [name]. If [name] are not doing the same for us, we are supporting them, and that's a good thing, but we're paying to make their lives better and they're not prepared to pay to make our lives better. There's a bit of a, I'm fixing someone's problem, mentality.”* – Participant 12
*“[…] Whereas most of them just think that system will work for us, then it's up to everybody else to cope, and often it involves doing extra work and extra expense. […] Along similar lines, we're finding now that local tertiary centres like the cardiology centre, the pl’stics, they're all developing their own portals and none of this is integrated a’ our end. I'm not sure whether they'll all be integrated at the other end’ither. They're all stand-alone systems and really, to me, they should be joined up.”* – Participant 15
**Subtheme 3: Data management-related barriers**
*“I think it's about ownership of an accountability for care delivery. So for your condition. Let's say you have a diagnosis. What are the associated actions to address that? Who owns them? Do you? Does your primary care provider? Does your secondary care provider? Being able to understand what other people have done in response to things. I think we can solve interoperability, it's a technical challenge’ big one. I'm still not sure it gets us to where we think it will, because I think you will persistently have separation of records. There will always be bits that we cannot share because they are not codable. Actually, interoperability is a—it's a really fancy workaround for creating a patient centred record.”* – Participant 12
*“I guess my framing is that we sit somewhere with this, between data and information and knowledge. If you achieve complete universal interoperability, you solve a data challenge. Healthcare and health delivery, generally, there is an element of nuance and it's your interpretation of it and what you're going to do with that piece of data, and how I have interpreted may be justifiably different to yours or someone else's interpretation, but the actions then hang from that. So I do not think a fully interoperable record will ever, in its own, get us passed that.”* – Participant 12
*“I think the junior ones [doctors] wouldn't be here for an extended period of time, so it may’e that they're not involved in going to that patient again, and they just keep repeating what they do. It will b’ people who've got a longer-term perspective who are following that patient through with their pregnancy, like me, who would be much more aware of it the importance of structuring data for the future users.”* – Participant 6
*“Now, in terms of data generation, as I say, I am fundamentally against the idea of putting an intermediary in there.”* – Participant 13
**Subtheme 4: Finance and business-related barriers**
*“Well, actually, this is a no-brainer. If you are not willing to have a free flow of data between our systems and the hospital systems, then you lose the contract.”* – Participant 4
*“In part, there's never been a financial incentive. Primary care probably have more potential to share data, because of their approach to structure. Secondary care has never been held to account on the quality or the value of i’s data that's collected. So we are in a payment-by-results world, I'm paid for delivering your care, none of the other stuff around it. […] I think there's never been a national financial incentive. So if we're looking to sign off a business case, no one has ever been held to account for whether this supports data sharing. Every business case has been inward focused, never outward focused.”* – Participant 12
*“We've always bought an American EPR, and they have a business model which has historically been lock you in, lock you in. So we're engaged with providers who are disincentivised to support it. We have never been given an incentive to challenge that.”* – Participant 12
*“Often working with these small companies, they will get things done in weeks, whereas when you're trying to do something with a big company like [EHR vendor name], that supply us with [EHR system name], you're talking about years. I think most of them won't have done it deliberately. […] Should there be some sort of national system that all these referrals can be plugged into? So that we can say to the suppliers, we could issue a ISM to say, 'You've got to be compatible with this system.'”* – Participant 15

#### Poor EHR systems usability

Most frontline healthcare providers reported negative experiences using EHRs to retrieve information from other organisations. Interoperability was described as being only able to share a narrow set of data rather than comprehensive records. Participants noted the need for external portals or multiple logins to access records from other organisations, though small hindrances alone, culminated in an incohesive user experience and a negative perception of efforts to achieve interoperability.

#### Institutional barriers

While healthcare providers are often frustrated with poor interoperability, they are seldom involved in improvement efforts. While the presence of the CCIO role does help to bridge the gap between clinical and administrative staff, participants often felt the scope of interoperability issues they faced are too great to be addressed with local resources.

Several CCIOs acknowledged that there is an underlying reluctance to cooperate with nearby facilities due to their unwillingness to make substantial changes to their own EHR systems. This is mostly due to fear of disruptive changes that might require retraining or disrupt existing workflows. Some perceived that their organisation may not benefit in an equal way when compared to other organisations involved, thus making the effort not worthy of the investment.

#### Data management-related barriers

Participants identified that effective inter-organisational information sharing relies on the creation of accurate, structured data by clinicians. Often, they felt that incentives to provide structured data varied widely between clinicians. Some participants felt clinicians who worked within the same organisation for a long period of time or were involved in the long-term care of patients (*e.g.,* in primary care) had greater incentives to invest their time in recording data to a higher standard. Some participants attributed variable data management to high clinical workloads and the lack of financial incentives for high-quality data management.

#### Finance and business-related barriers

Most participants indicated that there is often a lack of a clear business case for EHR system vendors to incorporate interoperability. Most systems are currently designed to inhibit data sharing with competing vendors’ EHR systems. Participants felt vendors appeared to be more content with using their existing systems in some hospitals to motivate neighbouring trusts to adopt the same system to achieve interoperability. Some participants, particularly those working in larger, urban trusts, suggested that coordinated procurement of the same EHR system by neighbouring trusts is the most pragmatic approach. Participants also pointed to a lack of a national plan to enhance interoperability through coordinated procurement of EHR systems.

### Future of EHR interoperability

Almost all participants described a future of tighter integration between disciplines where interoperability will continue to grow in importance. They described the growing desire to access clinical data generated by other providers across primary, secondary and community care settings, with some envisaging the opportunity to also write data to these record systems. Participant responses regarding the future of interoperability fell into four main subthemes (Table 6): (1) the need for common data standards, (2) to address existing EHR systems usability issues, (3) to incorporate patients in accessing their clinical records, and (4) the need to promote greater inter-organisational collaboration.

**Table 6:** Future of EHR Interoperability

**Subtheme 1: Need for common data standards**
*“I think the first step actually is the ability for all the various electronic health records just simply to be able to share their coded data and make it visible in the other clinical systems. So that's a first step, just to make the coded data available and visible, and searchable so that population health decisions can be made on the best data available I think. Alongside that will be quite a lot of work to improve the quality of that data and reduce variation or variability in coding between clinicians and organisations.” –* Participant 5
*“I think if you get patient view insight then you've got better way of knowing what to do with the data once you've got it. At the moment, it's very clinically focused and there's not enough patient-led in there. I think we, as clinicians, are privileged to look at patient's data and talk to them about their problems, but we should reflect that we are looking at patient data, and it's about the patient story, so we should be able to, we need to look at it from the patient angle. Does the data reflect who that patient is? A lot of the time, I don't think it does. It reflects what someone else's point of view is, from that specific specialty.”* – Participant 10
*“However, as we progress, I think we'd need to tailor it to that particular role to make it easier and faster for paramedics or, and secondary care, I know they would like to delve deeper into certain records. […] The same may happen for a care home nurse who generally doesn't need very much data, but at some point something is a bit more complicated, and then they can go and find out a little bit more. I think the hindrance is working out how to work that ethically, that any provide-, anyone who's trying to help the patient can go into deeper data. […] But who should be able to go further, and how do you give permission to those particular people in those particular circumstances, because that's quite complicated to work out.”* – Participant 10
*“So if we had a fully national interoperable approach to data, then I would be able to change the way I work, because I would be able to look at your record, in confidence, and know that is a single version of the truth. I therefore do not need to do all of these things. At the moment, because I do not have that trust, and no one has that trust, we look at it, but we still do the full A to Z assessment.”* – Participant 12
*“It will be worth it, there's no question about that, but the thought of trying to do that for everything, you think, is it going to be actually possible? This is where we really need something at national level that maybe forces all the IT suppliers to standardise in some way.”* – Participant 15
**Subtheme 2: Rethinking EHR systems design – usability, automation, and innovation**
*“I was talking to a patient the other day who said, 'I'm useless with computers', while using her mobile phone. I said, 'Well, you are using a computer. The reason is that the interface is so good, and the software is good, so it doesn't feel like a computer. It's just something that works for you', and that's how clinical software should be as far as possible.”* – Participant 6
*“My kind of vision of it would be that if the information were more held according to standards, that you could have another layer of software on the top as the user interface, but it would pull all the information from the relevant areas in a context specific way with AI to actually interrogate what knowledge was held without a human having to actually go and rummage around in all the different bits of software, because we've got over 100 different applications in our Trust.”* – Participant 6
*“It's going to be good design, so getting the specification right, trying to future-proof stuff as far as possible, using as generic an approach as possible, trying to create common interfaces so that people moving from one area to another don't get confused and everything looks very similar and familiar, and they're less likely to get it wrong. A lot of effort needs to be paid to user interface. A lot of them they're really poor I would say, too many mouse clicks, too many menus popping up, dropdowns, things that are occurring all over the place. If you look at the well-designed websites, you've got rollovers and cascading style sheets, and all kinds. I don't know the coding for stuff, but there's a lot you can do to make the interface more attractive and more initiative, and more dynamic. Having to click on things to open them to read them, what they say, instead of just being able to roll over and get an idea of what's in there, all these kinds of little things that are very commonplace now, they need to be built into EHRs as well to save time. A lot of thought needs to be given in terms of how the information is linked together, and how people might want to search it, but also with the flexibility to search it in novel ways that maybe weren't in the minds of designers.”* – Participant 6
*“I think the job is, then, to make the user experience of the system sufficiently good so that isn't too burdensome for the clinicians. That's where the ability of the system to automate routine and clerical tasks becomes very important. It's always going to be more difficult to type something into a computer than it is to just jot down a few notes on paper; always. The system only actually makes your life any easier if it does the routine boring stuff that it doesn't really take a healthcare professional to do, and that the machine can do.”* – Participant 13
*“[It] is not just about convenience, [it is] about building the system in such a way that it encourages or even forces you to be safe. It protects the patient from us making a simple mistake like that. Whereas, if you don't, you say it's up to the doctor, it's up to whoever to use it safely. We all make mistakes, we all can be busy, we can all be rushed, we can all be tired. To me, part of the responsibility when we put IT systems in is to try and prevent all of that. It is not about convenience. It's fundamental to patient safety.”* – Participant 15
**Subtheme 3: Patient involvement and contribution to health data**
*“I think our model previously was, 'These are the systems we need to do our job in the way that we want to do it,' but when you pivot things around and say, 'Actually, who are we really trying to serve?' It's the patients, and our citizens, and the taxpayer. Anything that would shift that focus would be really good from a patient point of view.”* – Participant 8
*“I think the other thing we need to do is we definitely need to pass the baton of care to the patients or to the citizen, rather than everyone just coming to hospital because that's the care they expect. That's where they expect to be treated. I don't think we can sustain that in the longer-term. […] There's a lot of data now that patients can collect themselves. I think it'd be incredible to use that in selected patients. That could help us in many, many ways, potentially; so, monitoring. People talk about devices, don't they? If you had a wearable that could monitor your vitals when you became unwell, you'd put this on. Then, if you were deteriorating, we could identify you sooner to do an intervention and that intervention could be very simple. […] In terms of our interventions, I think, we'd definitely use technology and selected patients to help them manage themselves better.”* – Participant 8
*“I think the clinical systems need to be open to the patient so that they can control, they can see what's going on with their data. It's written for patients about them, so I think the focus needs to be changed a bit more about that, in that direction. I think that's where, without getting the standard to join up what's already in there, it's quite difficult to do that.”* – Participant 10
*“I think the first change is just really getting the data out of other people's systems at the moment. You need to get the secondary care data up and going, you need to get the social care's data up and going. You need to get patients on board and working what they want, where their view, what's their vision of their system, how do they imagine their clinical records to look from an interoperable side?”* – Participant 10
*“What I would like to see is I would like to be able – say, in terms of interoperability, I want to be able to see what the patient's contributed, I'd like to be able to see—at the moment I don't have any patient contribution to any of my record. Actually, I believe the data is all theirs, not mine.” –* Participant 1
*“It's going to sound absolutely ridiculous, but I think it's about ownership of an accountability for care delivery. So for your condition. Let's say you have a diagnosis of blah-blah-blah. What are the associated actions to address that? Who owns them? Do you? Does your primary care provider? Does your secondary care provider? […] it's my record and you are allowed to have a look in it, but I own it and it follows me, as a patient. I think we can solve interoperability, it's a technical challenge-, big one. I'm still not sure it gets us to where we think it will, because I think you will persistently have separation of records. There will always be bits that we cannot share, because they are not codable. Actually, interoperability is a—it's a really fancy workaround for creating a patient-centred record.”* – Participant 12
**Subtheme 4: Greater inter-organisational collaboration**
*“Interoperability, it would be the ability to move information between the systems, whilst retaining its structure and all associated metadata. I want my blood pressure to look the same in both systems, I'll want to know it was a seated rather than a standing blood pressure, or something like that.”-*Participant 12
*“What I would hope would happen is that, actually, we would begin to realise that flow of data between systems, and that should be all important. A lot of it is the publicity in getting it to our politicians and various other things. Rather than saying there's a data breach, where my data was wherever it was, it's to actually get across to that level, to say, 'Actually, this is really important.' If your data is shared between all the various people who are part of your care, it can only improve your care. Actually, those patients want to help progress. They want their condition and what you learn about it, to help others, but you can't do that if you're working in silos.”* – Participant 4
*“There is a problem, so from our point of view, it is probably saying, 'Well, if you want to bring in a new system, it has to be interoperable with our [vendor name] EHR.' This is the main source of truth, so it has to be interoperable with that. You probably won't make an enormous amount of friends all the time because a lot of people will say, 'Well, this company doesn't want to do it, [vendor name] doesn't want to do it, and now I can't have my use in that.'” –* Participant 4
*“The other problem is I think, you don't always know what other organisations are already doing. The projects aren't being, aren't transparent enough, and you can't see what the user requests are for each project, what's their expectation of how it should work, and there's probably a significant overlap which most, a lot of organisations could, are just duplicating work… there doesn't seem to be a way of viewing what projects are ongoing or what stage they're at, who's involved with them, who to connect to, to talk about them. Or if you see a certain issue, or if you do this, then you're taking this out, and did you realise our workflow was dependent on that?” –* Participant 10

#### Need for common data standards

Implementing data standards was a near-universal need highlighted by study participants. The implementation and adherence to standards should not be limited to immediate clinical vocabulary found within EHRs but needs to include communication tools between providers, mechanisms for patient consent, and built-in for new technologies to be added in the future.

#### Rethinking EHR systems design: usability, automation, and innovation

Participants indicated that greater application of user-centric design principles is required to improve interoperability. With more intuitive user interfaces that are better aligned with clinician workflows, user errors, and risks to patient safety can be potentially minimised.

Participants described the potential for the use of novel technologies including artificial intelligence to automate manual data entry processes and remove administrative burdens currently required to improve interoperability. Other potential benefits of automation include improving care timeliness, data accuracy, and overall quality and relevance of clinical information in EHRs.

#### Patient involvement and contribution to health data

Participants identified the need to engage patients in the management of their own clinical data. This was perceived to both improve EHR data reliability, facilitate better communication between clinicians and patients, and empower patients to have more control over their own care. Other indirect benefits postulated included the reduction of workload for healthcare providers by enabling remote monitoring of patients with smart devices integrated into EHRs and reducing the need to repeat investigations and history taking due to incomplete or inaccurate documentation found within the EHRs.

Participants identified that making EHRs accessible to patients would require a significant redesign of EHR interfaces, but many felt these improvements could also make systems more useable for clinicians. While most participants supported patients having greater access to their own data, opinions differed as to whether this should include the opportunity to write into their own records.

#### Greater inter-organisational collaboration

Participants identified greater inter-organisational collaboration as a key aspect for the future of interoperability and emphasised the importance and value of coordinating tasks and allocating appropriate resources between organisations. Similarly, the currently often duplicative EHR improvement efforts happening across organisations could be minimised by having greater visibility and alignment of similar initiatives already taking place nearby.

## Discussion

### Summary of principal results

At present, EHR interoperability across NHS facilities remains largely intra-organisational, patchy, and incomplete. The interviewed CCIOs demonstrated a rather narrow interpretation of the various types of levels of interoperability and its perceived value, with captured views primarily focussed on information sharing only within their own immediate healthcare setting and/or with nearby GPs. Additional information sharing with private service providers, or incorporating patient-generated information, social care, or welfare service providers, did not seem to factor into CCIOs’ perception of the value of greater interoperability.

Initiatives aimed at improving interoperability are gradually developing, but largely remain limited in scope, sophistication, and prevalence. CCIOs are often hindered by the introduction of solutions in a piecemeal, uncoordinated fashion, and without the support and vision necessary to coherently improve EHR interoperability at regional or national levels.

Limited EHR interoperability has important patient safety implications. The inability to easily access and transfer data into a unified source contributes to data fragmentation and lack of clarity on patients' overall health status. This results in suboptimal care planning and coordination, duplication of efforts, and more defensive practice. However, a minority of participants also expressed opposing views, noting that poor interoperability posed a threat to clinician productivity rather than to patient safety directly.

Our study demonstrated that the advantages of enabling greater interoperability are evident and commonplace in the NHS. The widespread recognition of the need for interoperability amongst healthcare workers, patient expectations, and the centralised structure of the NHS itself, were all identified to be facilitators to further encouraging future interoperability efforts both within and across facilities. Challenges noted include poor usability of many EHR systems in service, as well as various institutional, financial, and data management-related barriers.

Looking ahead, however, most participants expressed a positive outlook regarding the future of interoperability in the NHS. The mandating of common data standards and interoperability functionality in procurement contracts, a greater focus on improving EHR systems design and usability with an emphasis on interoperability from the outset, strengthening the role of patients concerning their own health data within EHRs, and tackling inter-organisational collaboration barriers, were all main themes highlighted.

### Strengths & limitations

To the best of our knowledge, this is the first study to investigate thoroughly the views and perceptions of NHS healthcare administrators with clinical roles surrounding the topic of interoperability and patient safety in relation to EHR implementation and use. The interviewed CCIOs came from a diverse range of clinical backgrounds working in NHS facilities across England. The interview topic guide evaluated the subject comprehensively and was developed by a multidisciplinary team based on evidence found in the wider literature. The overall study design was done in accordance with the COREQ best practice guideline [44]. The findings were coded by two qualitative researchers with backgrounds in clinical medicine, public health, and patient safety.

However, our findings must be interpreted in the context of certain limitations. Firstly, the recruitment method employed did not allow for a representative sample of CCIOs across the whole of England to be obtained. Self-selection bias is expected to be present as CCIOs agreeing to participate are likely those who are either more enthusiastic about research or have more controversial views regarding the topic and are more willing to share it. The relatively small sample size overall may also impact the external validity of our findings given the varied clinical environments found across England and other high-income countries. The inability to interview participants in person due to COVID-19 restrictions may also negatively impact the quality/richness of their responses and present some level of recall bias. However, this may be offset by its convenience and some participants finding it less intimidating to be interviewed remotely and thus more able to speak freely. Lastly, the shifting needs resulting from the COVID-19 pandemic may have both influenced what informed participants’ perceptions of the EHRs described during our interviews, as well as what may have been omitted.

### Comparison with prior work

Many of the themes and subthemes identified by our study participants mirror findings from the wider EHR literature.

The advantages of using interoperable EHRs are well-documented [8, 29, 45–47]. While many studies pertained to clinical staff, the findings were largely in line with benefits recognised by CCIOs in our study as well [19]. Primary benefits included greater healthcare provider productivity, communication & care plan coordination between providers, and enhancing overall care quality & safety [8, 36, 45, 47]. In addition to immediate clinical uses, there are also secondary benefits identified in the literature [46–48]. *Sandhu *et al*.,* highlighted the value of EHRs to public health (*e.g.,* communicable disease surveillance), care quality management, medication & device safety, optimising of health systems management and notably, clinical research [49]. *Nordo *et al*.,* highlighted that the use of EHR data has been demonstrated to *“streamline clinical research processes at health institutions, improve data quality by reducing the number of transcription errors, support the evaluation of research protocols feasibility and increase the availability of patients to participate in research”* [46, 50]. The authors also remarked, however, that the true research potential possible was curtailed by limited interoperability [46].

Likewise, many of the barriers to achieving greater EHR interoperability described by our study participants were also not entirely unexpected. For example, the problem of poor usability of EHR systems corroborates with findings from existing studies [51, 52]. A recent publication by *Adams *et al*.*, concerning computerised provider order entry (CPOE) systems used alongside EHRs in many hospital settings, found that usability problems contributed to nearly 97% of medication-related errors, with data entry and workflow support being the two most common types of usability issues [53]. Similarly, other design limitations such as the failure to support clinical workflow and unresponsive/slow systems have also been previously recognised [54–56]. In a systematic review by *Mello *et al*.,* the lack of consensus regarding terminology, classification, communication, and data transfer standards, all well-recognised problems, remain notable hurdles to interoperability today [17].

Many of the suggested solutions mentioned by our participants have also been proposed in one form or another in the existing literature. Prioritising EHR interoperability from the outset, mandating data exchange standards, promoting user-centric design & systems usability, discouraging vendors from establishing proprietary data exchange networks, and the involvement of end-users in the design and implementation process, were all concepts previously identified [17, 57–59]. At the organisational level, this can range from providing greater financial incentives for inter-organisational collaboration and reducing competing interests to cooperation, to simply improving communication and raising awareness of similar HIT initiatives nearby [17, 60]. The growing recognition of the importance of increasing patient involvement in their EHR data was also borne out by the recently updated NHS guidance detailing the new channels with which patients can access their GP records [61].

### Implications for policy and future research

Past attempts by various stakeholders independently trying to solve interoperability through incremental measures such as developing new portals to remotely access a limited set of clinical parameters, typically do not solve the problem of poor interoperability and seldom lead to a meaningful improvement in patient safety. Instead, there needs to be a concerted effort at an individual, technical, organisational, and even national level to bring about meaningful EHR interoperability in the NHS.

At the individual healthcare provider level, formalised teaching on EHR usage starting in their undergraduate medical education and refresher training throughout their residency years may help instil a more standardised approach to handling healthcare data amongst the profession, akin to other basic skills such as history-taking and prescription writing. From the technical perspective, EHRs will likely benefit from leveraging human factors expertise and incorporating greater clinician feedback to better align EHRs with evolved user needs and expectations. At the organisational level, policies aimed at raising awareness of HIT implementation initiatives in nearby trusts would help mitigate the tendency for siloing, reduce duplicate costs/efforts, and culminate in more a coordinated, coherent implementation of HIT systems. A simultaneous re-evaluation of existing policies which may inadvertently perpetuate perverse incentives to hoard clinical data and generate artificial barriers, such as performance metrics for individual trusts, is also necessary to cultivate an environment for collaborative efforts to take place. At the greater regional or national level, mandating the use of common data standards for interoperability at the point of EHR procurement and renewal is key – preferably well-established ones already validated and in use in other countries’ healthcare systems. The fact that many providers are often ‘locked in’ to a particular vendor’s products will likely require regulatory intervention and financial incentives to discourage such business practices. Health systems from countries with smaller populations have demonstrated how this could potentially be achieved. In Finland for example, legislation concerning EHR products has mandated common interoperability standards, testing, and certification processes [62]. Relevant health information systems and exchanges were incrementally rolled out since 2010, culminating in data being seamlessly transferrable between healthcare providers, pharmacies, and patient-accessible EHRs [62]. A similar approach can perhaps be adapted for the NHS context in future EHR interoperability improvement endeavours.

Existing EHR research has often centred on outcome measures such as adoption rates, potential cost/time-savings, or end-user issues such as usability and convenience [51, 55, 63–67]. While these efforts were valuable in monitoring EHR implementation progress, our study has highlighted the importance of following up with studies which capture the quality of such efforts and the practical challenges confronted by other health systems stakeholders. As demonstrated, some barriers may not appear in key performance indicator metrics or are easily quantifiable, but nonetheless are present across many healthcare settings and influential to the success or failure of implementing interoperable EHRs and realising their purported benefits. Future research efforts must be devoted to other healthcare workers involved along a patient’s care pathway, EHR system vendors, policymakers, and patients & caregivers themselves. Better understanding these practical barriers can help inform the development of more relevant and effectual HIT policies and hasten the realisation of interoperability in a more cohesive manner.

## Conclusion

Despite the growing prevalence of EHRs in the NHS, interoperability between systems across different healthcare settings and providers remains suboptimal. While numerous and convoluted, barriers are primarily not technical but rather institutional or business-sided (*i.e*., lack of sufficient political support, unclear national data standards, inadequate development of robust health information exchanges, and insufficient financial incentives to prioritise EHR interoperability).

Solutions will likely require a concerted effort from a multitude of approaches. Future efforts must focus on mandating the implementation of common data standards, tackling systems usability issues with end-users, and taking into greater consideration the growing role of patients and their ability to access and contribute to their own health information found within EHRs.

### Supplementary Information

**Additional file 1:** **Supplement 1.** Topic Guide.

## Data Availability

Data is available upon reasonable request to the corresponding author.

## Abbreviations

A&E: Accident and emergency
CCIO: Chief clinical information officer
CPOE: Computerised provider order entry
EHR: Electronic health records
EMT: Emergency medical technician
FHIR: Fast Healthcare Interoperability Resources
GP: General practitioner
HIT: Health information technology
ICS: Integrated care system
ICT: Information and communications technology
NHS: National Health Service
