# Supplementary material for: Perceptions of chief clinical information officers on the state of electronic health records systems interoperability in NHS England: a qualitative interview study
Source: BMC Med Inform Decis Mak. 2023 Aug 12;23:158. doi: 10.1186/s12911-023-02255-8 (PMC10423420; doi:10.1186/s12911-023-02255-8)
Supplement: Supplementary file 1 — Additional file 1: Supplement 1. Topic Guide. [file 12911_2023_2255_MOESM1_ESM.pdf]

Document version: 1.7
Document date 02/11/2020
ICREC ID: 20IC5906

Supplement 1: Topic Guide

Participant ID Number:	Gender: Male / Female
Date (DD/MM/YYYY):	

Project title: The perceptions of chief clinical information officers on the state of electronic health records systems interoperability in England

Going over the Patient Information Sheet & Informed Consent:

- Overview and purpose of study
- Aims of interview and expected duration
- Who is involved?
- Why participant's involvement is important, advantages and disadvantages
- What will happen to the results of this study?
- Any questions?
- Completion of informed consent form

Summary of study objectives: Before I ask you further questions, here is what we will be covering in today's interview:

1. To explore the perceptions and expectations of chief clinical information officers (CCIO) regarding the current state of EHR interoperability and its effects on patient safety in hospitals.
2. To investigate past facilitators and barriers to achieving interoperability in the past decade during the initial introduction of EHR systems into the NHS.
3. To explore how CCIOs perceive the continued development and evolution of EHR interoperability would improve patient safety in the NHS for the coming decade.

Document version: 1.7
Document date 02/11/2020
ICREC ID: 20IC5906
Participant Information: May I ask you some details about you and your background as a healthcare systems administrator/chief clinical information officer?

**Job Title**	
For how long have you been working as a chief clinical information officer?	
Do you work in an urban/rural catchment area?	
How many other hospitals/GP practices are in the area serving the same patient population?	
How long has your hospital been using electronic health records?	
What EHR vendor's system is currently in use at your present workplace?	

**Aim(s)**	**Topics & Prompts**	**Examples/Clarifications**
To investigate the perceptions and expectations of chief clinical information officers (CCIO) regarding the current state of EHR interoperability and its effects on patient safety in hospitals.	1. **In your opinion, how would you define EHR and EHR interoperability?**<br>2. **How would you describe the current state of EHR interoperability in your hospital/healthcare facility?**<br>3. **How do you feel the current state of EHR interoperability impacts patient safety in NHS hospitals? Can you give any examples?**<br>4. **In your department, what is your perception of the clinical culture towards the EHR systems currently in use and the issue of interoperability? Can you elaborate?**<br>5. **What factors do you feel help or hinder the adoption of changes necessary to have interoperable EHRs at your workplace?**	• EHRs are defined as: computer software that physicians use to track all aspects of patient care. Typically, this broader term also encompasses the practice management functions of billing, scheduling, etc.<br>• INTEROPERABILITY is defined as: the ability of health information systems to work together within and across organisation boundaries to advance effective delivery of healthcare for individuals and communities.<br>• FACILITATORS: Factors that improved EHR interoperability/mitigated potential problems

		• BARRIERS: Factors that were detrimental to achieving EHR interoperability
To investigate past facilitators and barriers to achieving interoperability in the past decade during the initial introduction of EHR systems into the NHS.	**1. EHRs have been available in most NHS trusts for over a decade now. Looking back, what factors do you think contributed to the state of EHR interoperability you have described earlier?**<br>• What do you think led to the facilitators/barriers you have mentioned earlier?<br><br>**2. What implementation strategies used during the introduction of EHRs were helpful in promoting interoperable EHRs? What detracted from this?**<br><br>**3. In some hospitals where EHRs have been introduced, interoperability with other healthcare facilities nearby often remains limited. What could be the reasons for this?**<br>• Administrative problems?<br>• Technical problems?<br>• Contextual problems?<br>• Resource problems?<br><br>**4. What is or could be your role in deciding whether or not to introduce changes that affect EHR systems and interoperability?**	
To explore how CCIOs perceive the evolution and development of EHR interoperability would improve patient safety in the NHS for the coming decade.	**1. How do you see the area of EHR interoperability develop over the next decade?**<br><br>**2. Do you think there is a need for change in EHR systems to address interoperability to improve patient safety in the NHS? If so, what sort of changes do you think are needed?**<br><br>**3. Are there any changes in your immediate workplace/healthcare facility that you think can help improve EHR interoperability?**<br>• Across surrounding healthcare facilities?<br>• Across the NHS itself?	

	**4. Are there to solutions addressing EHR interoperability from other health systems around the world which can be adopted for use in the NHS context? If so, can you elaborate?**<br><br>**5. Is there anything from the current COVID-19 pandemic which can learned and utilised to improve EHR interoperability?**	

Closing: Is there anything else that you think is important about using electronic health records, interoperability, and patient safety in NHS trusts that we have not talked about?

- Summarize covered domains
- Any other questions/concerns?
- Ensure participant has copies of participant information sheet and consent forms; provide any additional information as needed
- Thank interviewee for their input and participation.
